# Crovalimab Rescue Therapy in a Case With Genetic Complement Mediated Thrombotic Microangiopathy

**DOI:** 10.1016/j.xkme.2025.101185

**Published:** 2025-11-10

**Authors:** Christof Aigner, Zoltán Prohászka, Ágnes Szilágyi, Georg A. Böhmig, Klaus Arbeiter, Alice Schmidt, Gere Sunder-Plassmann

**Affiliations:** 1 Division of Nephrology and Dialysis, Department of Medicine III, Medical University of Vienna, Vienna, Austria; 2 Research Laboratory, Department of Internal Medicine and Haematology, Semmelweis University, Budapest, Hungary; 3 Division of Pediatric Nephrology and Gastroenterology, Department of Pediatrics, Medical University of Vienna, Vienna, Austria

**Keywords:** Atypical hemolytic uremic syndrome, complement system, crovalimab, genetic renal disease, ravulizumab, thrombotic microangiopathy

## Abstract

Complement mediated thrombotic microangiopathy (C-TMA) is a rare disease resulting in kidney failure and other organ manifestations. Current treatments include the complement C5 blockers eculizumab and ravulizumab as well as plasma therapy. We report on a young adult man with a long-standing history of genetic C-TMA (GC-TMA) because of a likely pathogenic missense variant in *CFH*. After several years without clinical signs of TMA and normal kidney function (CKD G1A2), without recent specific therapies, he presented with acute kidney injury, microangiopathic hemolysis, and nephrotic range proteinuria. Plasma therapy and ravulizumab failed to stop hemolysis, and he commenced kidney replacement therapy 11 days after admission. Laboratory analyses disclosed suboptimal complement inhibition and low free ravulizumab serum concentrations. Four weeks after admission, we started treatment with crovalimab, a novel humanized anti-C5 antibody. Hemolysis improved immediately and kidney function recovered after 3 months of dialysis treatment and improved continuously during 1 year of therapy with crovalimab. The excellent and rapid response to crovalimab potentially suggests that the engineering of crovalimab, facilitating also subcutaneous administration, may result in a different pharmacokinetic and pharmacodynamic profile of crovalimab as compared with standard C5 inhibitors in patient with nephrotic range proteinuria.

Uncontrolled activation of the complement system plays a causal role in complement mediated thrombotic microangiopathy (C-TMA) and C3 glomerulopathy and also contributes to injury in numerous other kidney diseases.^1^^,^^2^ Fortunately, the last years have witnessed the clinical development of an evolving number of complement modulators beneath the well-established complement C5 blockers eculizumab and ravulizumab, which is engineered from eculizumab, resulting in a molecule that targets the same epitope as eculizumab with an extended half-life.^3^, ^4^, ^5^ One of these molecules is crovalimab, a novel humanized anti-C5 antibody, that is successfully used subcutaneously in C5 inhibitor naive and pretreated patients with paroxysmal nocturnal hematuria.^6^ Crovalimab is engineered using the sequential monoclonal antibody recycling technology-immunoglobulin. This technique allows for combined isoelectric point-, neonatal Fc receptor-, and pH-dependent affinity engineering to prolong antibody half-life in the plasma and reduce the drug dosage.^7^ Crovalimab further binds to the C5 β-chain, which is a different epitope from eculizumab and ravulizumab. It thus inhibits C5 activation in patients carrying the *C5* variant p.(Arg885His), p.(Arg885Ser), or p.(Arg885Cys) located in the C5 α-chain, which confer resistance to eculizumab and ravulizumab.^8^, ^9^, ^10^, ^11^

We report, to the best of our knowledge, the first case of genetic C-TMA (GC-TMA) that responded to treatment with crovalimab after failure of ravulizumab and plasma therapy.

## Case Report

A 23-year-old man of Asian descent presented to the emergency department with a history of fever since about 2 weeks, headache, and weakness in the legs. His height was 1.80 m, and his body weight 60 kg. His blood pressure was 150 over 100 mm Hg, with normal heart rate, body temperature, and peripheral oxygen saturation. The physical examination was unremarkable except for peripheral edema. Laboratory analysis disclosed kidney failure (serum creatinine 7.59 mg/dL) and microangiopathic hemolytic anemia with a hemoglobin concentration of 6.8 g/dL, a platelet count of 147 G/L, a lactate dehydrogenase concentration of 947 U/L, and schistocytes in the peripheral blood smear. The protein to creatinine ratio in the urine output was 9,938 mg/g and the urine dipstick was positive for hemoglobin (>1 g/dL) with 171 red blood cells per μL urine output.

The clinical diagnosis was relapse of familial GC-TMA having had a first flare at the age of 3 years that responded well to plasma exchange as did 3 further flares in the same year. He then received 2 units of fresh frozen plasma every 8 weeks until the age of 14 years. During the years to come he had regular health check-ups that showed normal kidney function without overt proteinuria including his last laboratory tests 4 months before his current admission (serum creatinine 0.99 mg/dL, urine albumin to creatinine ratio 44 mg/g).

Genetic testing a couple of years ago showed the presence of a heterozygous, likely pathogenic variant in *CFH*, p.(Arg1182Ser), ClinVar-ID 988141, which was inherited from the paternal grandmother with end-stage kidney disease because of GC-TMA (the father, who has no history of TMA, refused genetic testing).

Within a few hours after he now presented in the emergency room, he received a plasma exchange with fresh frozen plasma (FFP) and then a first dose of ravulizumab (2,700 mg) within 24 hours after admission. Because kidney function and platelet count declined with ongoing hemolysis, daily plasma infusions were given from day 5 until day 11 when he commenced kidney replacement therapy and received a second dose of ravulizumab (600 mg). Furthermore, on day 14, the patient received the next scheduled dose (3,300 mg). Because the clinical diagnosis seemed clear, we refrained from performing a kidney biopsy.

Evaluation of complement inhibition in 2 blood samples (one 10 days after the initial ravulizumab dose and one taken 3 days after the second full dose) showed suboptimal complement inhibition as indicated by nondeficient activity of the classical and alternative pathways and a low free ravulizumab serum concentration in both samples (36 and 28 μg/mL, respectively; Table 1).^4^ All the laboratory essays included in Table 1 are measured in the laboratory of Zoltán Prohászka in Budapest, Hungary.^4^^,^^12^

**Table 1:** Changes in Complement Profile During Complement Inhibitory Treatment

Days after start of GC-TMA episode	11^a^	14^b^	30^c^	34^d^	157^e^	360^e^	Reference range
TCA, classical pathway	58	31	62	13	25	6	48-103 CH50/mL
TCA, alternative pathway	24	23	75	1	1	1	70% to 125%
Complement C3	1.14	0.87	0.66	0.9	0.92	1.17	0.9-1.8 g/L
Complement C4	0.42	0.3	0.15	0.49	0.44	0.48	0.15-0.55 g/L
Factor H antigen	838	697	329	751	683	1224	250-880 mg/L
Complement factor I antigen	112	75	93	93	79	92	70% to 130%
Complement factor B antigen	144	79	73	n.a.	77	84	70% to 130%
Anti-factor H IgG autoantibody	81	86	n.a.	n.a.	n.a.	n.a.	<110 AU/mL
sC5b-9 (TCC)	355	277	379	1041	214	341	110-252 ng/mL
C1q antigen	111	72	112	140	334^6^	309^6^	60-180 mg/L^f^
Haptoglobin	0.15	<0.07	0.56	0.34	1.03	2.09	0.3-2.0 g/L
Serum free ravulizumab	36	28	1	2	n.a.	n.a.	μg/mL^g^

Abbreviations: n.a., not available; TCA, total complement activity; TCC, terminal complement complex.

a Sample taken 10 days after first dose of ravulizumab (presumably heavily influenced by plasma therapy).

b Sample taken 3 days after second dose of ravulizumab and 2 days after last fresh frozen plasma infusion (presumably also influenced by plasma therapy). The patient received an additional dose of 600 mg of ravulizumab on day 11 after the loading dose and received 2 FFP on day 12 but none on days 13 and 14.

c Sample taken after 12 sessions of plasma exchange in 15 days and before the first dose of crovalimab.

d Sample taken 4 days after first dose of crovalimab (1,000 mg intravenously on day one and 340 mg subcutaneously on the subsequent day).

e Sample taken during crovalimab therapy.

f For time points 157 and 360, ref. range 150-320 mg/L.

g Trough levels <50 μg/mL are considered subtherapeutic.^4^

Because treatment with ravulizumab failed to stop hemolysis, the patient then received daily plasma-exchanges with FFP for 14 days. The signs of hemolysis improved somewhat, and the platelet count stabilized ∼100 G/L. However, plasma therapy also did not improve kidney function.

Eventually, one month after admission, the patient was given the first off-label dose of 10,00 mg crovalimab intravenously followed by 340 mg subcutaneously on the next day as well as on days 8, 15, and 22. Of note, at the time of the initiation of treatment with crovalimab, the patient showed persistent nephrotic range proteinuria with a urine protein to creatinine ratio of 3,959 mg/g. Treatment was continued with a dose of 680 mg on day 29 and every 4 weeks thereafter.

**Figure 1:**
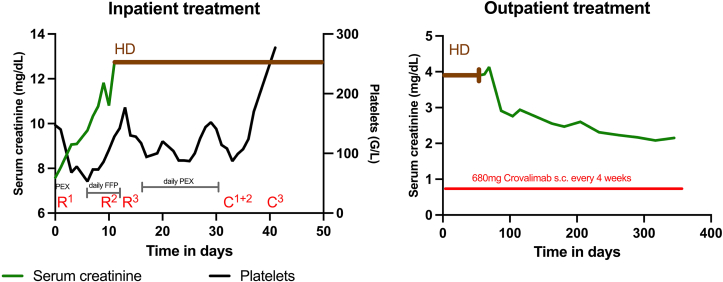
Inpatient and outpatient course of laboratory values and anti-C5 therapies. Abbreviations: C, crovalimab; FFP, infusion of fresh frozen plasma; HD, hemodialysis; PEX, plasma exchange; R, ravulizumab.

Six days after the first dose of crovalimab, the platelet count increased into the reference range (170 G/L) and the serum lactate dehydrogenase concentration returned to normal some 3 weeks later. Kidney function, however, did not improve immediately. Laboratory findings, anticomplement therapies, and kidney replacement therapy during the hospital stay are illustrated in Fig 1. Subsequent genetic testing ruled out the presence of the p.(Arg885His), p.(Arg885Ser), or p.(Arg885Cys) SNPs located in the C5 α-chain.

Following discharge from the hospital after 36 days he continued to receive 680 mg of crovalimab every 4 weeks, and we discontinued dialysis treatment after about 3 months. Evaluation of complement inhibition during crovalimab therapy showed low activity of both classical and alternative complement pathway markers and low levels of the soluble terminal complement complex, indicating a well-controlled alternative complement pathway (Table 1).^4^ Approximately 12 months after the GC-TMA relapse, the patient continued to show further improvement of his estimated glomerular filtration rate (Chronic Kidney Disease Epidemiology Collaboration [CKD-EPI] 2021) to 43 mL/min/1.73 m^2^ with a urine protein to creatinine ratio of 1,058 mg/g (CKD stage G3bA3). The time course of the outpatient laboratory results and therapies is shown in Fig 1.

## Discussion

Therapy with either eculizumab or ravulizumab represents the current standard of care for patients with C-TMA. Hematologic response and improvement of kidney function are usually achieved within the first week(s) of treatment.^4^^,^^13^^,^^14^ One important study showed a 5-year cumulative incidence of kidney failure free survival of 85.5% compared with 39.5% for a genotype matched control population. As such, one case of kidney failure is prevented for each 2.17 patients treated. Nevertheless, outcomes are suboptimal. In addition, about 20% of patients still present with irrecoverable kidney function, and some 30% have CKD stages 3-5 after 6 months.^14^^,^^15^

Reasons for lack of efficacy of eculizumab or ravulizumab include the presence of distinct genetic variants in the α-chain of C5, heavy proteinuria with loss of anti-C5 antibodies in the urine, higher serum creatinine at presentation, older age, high systolic blood pressure at presentation and a longer time gap from presentation to C5 inhibition.^8^^,^^15^, ^16^, ^17^ Of note, neutralizing antiravulizumab antibodies have not been reported so far.^18^ Thus, nephrotic range proteinuria in our case may have contributed to the lack of efficacy of ravulizumab by losses into the urine, as it was recently shown for eculizumab.^16^^,^^17^ Some data suggest that the initial dosing regimen might result in a suboptimal complement blockade in certain patients and suggest giving a higher initial dose. However, this was only shown for eculizumab but might also be conceivable for patients treated with ravulizumab.^19^

Intensive plasma therapy, which is considered less effective as compared with C5 inhibition, although it had been effective in the past, also failed to overcome the alternative complement pathway activation in our case. Another important point of concern is the potential presence of genetic variants in the C5 α-chain that confer resistance to eculizumab and ravulizumab, primarily in patients of Asian ancestry.^8^, ^9^, ^10^, ^11^ Sanger sequencing, however, did not disclose any genetic variant in the C5 α-chain of our patient.

As shown in our case, the excellent and rapid response to C5 inhibition using crovalimab may suggest that the engineering of crovalimab, facilitating also subcutaneous administration, may result in a different pharmacokinetic and pharmacodynamic profile of crovalimab as compared to standard C5 inhibitors in patient with nephrotic range proteinuria. In addition to the recycling technology, crovalimab uses isoelectric point engineering to facilitate rapid receptor-independent uptake via endocytosis, thereby binding and eliminating more, free C5 at lower doses. Furthermore, crovalimab uniquely inhibits C5b9 deposition on membranes, further limiting membrane attack complex-mediated tissue damage.^6^^,^^7^ However, we cannot rule out an effect of plasma therapy on ravulizumab in this patient, because additional doses of ravulizumab were not administered after every plasma infusion. Our reasons for switching to an alternative C5 inhibitor rather than to eculizumab was the assumption that the patient might carry the genetic variant in the α-chain of C5. Another fact to consider is that cell lysis-based assays for both classical and alternative pathway activity determination are not reliable in the case of ravulizumab because of the pH-dependent binding of ravulizumab to C5. However, the alternative pathway testing we used is enzyme-linked immunosorbent assay based and therefore together with the sC5b9 essay can be used to determine effectivity of complement inhibition by ravulizumab.

Importantly, kidney function continuously improved in our patient, even after 1 year of therapy with crovalimab. We are now curious about the results of the ongoing phase 3 trial of crovalimab in patients with C-TMA, specifically in cases presenting with heavy proteinuria.
